# Increased Risk of Postthoracotomy Pain Syndrome in Patients with Prolonged Hospitalization and Increased Postoperative Opioid Use

**DOI:** 10.1155/2016/7945145

**Published:** 2016-06-02

**Authors:** Michelle A. O. Kinney, Adam K. Jacob, Melissa A. Passe, Carlos B. Mantilla

**Affiliations:** ^1^Department of Anesthesiology, Mayo Clinic, 200 First Street SW, Rochester, MN 55905, USA; ^2^Department of Anesthesiology, Anesthesia Clinical Research Unit, Mayo Clinic, 200 First Street SW, Rochester, MN 55905, USA

## Abstract

*Background.* Postthoracotomy pain syndrome (PTPS) is unfortunately very common following thoracotomy and results in decreased quality of life. The purpose of this retrospective study was to determine perioperative patient, surgical, and analgesic characteristics associated with the development of PTPS.* Methods.* Sixty-six patients who presented to the Mayo Clinic Rochester Pain Clinic were diagnosed with PTPS 2 months or more after thoracotomy with postoperative epidural analgesia. These patients were matched with sixty-six control patients who underwent thoracotomy with postoperative epidural analgesia and were never diagnosed with PTPS.* Results.* Median (IQR) hospital stay was significantly different between control patients (5 days (4, 6)) compared with PTPS patients (6 days (5, 8)), *P* < 0.02. The total opioid equivalent utilized in oral morphine equivalents in milligrams for the first three days postoperatively was significantly different between control patients and PTPS patients. The median (IQR) total opioid equivalent utilized was 237 (73, 508) for controls and 366 (116, 874) for PTPS patients (*P* < 0.005).* Conclusion.* Patients with a prolonged hospital stay after thoracotomy were at an increased risk of developing PTPS, and this is a novel finding. Patients who utilize higher oral morphine equivalents for the first 3 days were also at increased risk for PTPS.

## 1. Introduction

Postthoracotomy pain syndrome (PTPS) is unfortunately very common. In a recent prospective trial, we determined that 68% of patients had pain three months following serratus-sparing posterolateral thoracotomy or limited thoracotomy, resulting in decreased quality of life, physical function, and vitality [1]. We have also demonstrated that preoperative gabapentin in the setting of multimodal analgesia with thoracic epidural bupivacaine and hydromorphone, along with acetaminophen, ketorolac, and intravenous fentanyl via patient-controlled analgesic (PCA pump), did not decrease acute or chronic postthoracotomy pain [2].

Various studies have made observations about predictive risk factors for PTPS. In a prospective study by Ochroch and colleagues involving 120 patients, it was demonstrated that women reported more pain than men during their hospitalization and for 48 weeks postoperatively [3]. In a separate prospective study by Ochroch and colleagues, the initiation of thoracic epidural analgesia prior to thoracotomy incision rather than at the time of rib approximation was not found to significantly decrease acute pain during hospitalization or at 48 weeks [4]. A critical review of the literature by Wildgaard et al. concluded that intercostal nerve injury is probably the most important risk factor in the development of chronic postthoracotomy pain [5]. A systematic review of randomized trials by Joshi and colleagues noted that the efficacy of thoracic epidural combining local anesthetic and opioid is established for decreasing acute postthoracotomy pain, but it remains unclear if perioperative analgesia is related to the development of PTPS [6]. Therefore, the purpose of this retrospective, case-control study was to determine perioperative characteristics for the development of PTPS.

## 2. Materials and Methods

Institutional Review Board approval was obtained prior to reviewing medical records. The Mayo Clinic Life Sciences System database was searched using CPT codes 32035 through 32525 to identify all patients who had undergone a noncardiac thoracic procedure from May 14, 2000, through December 31, 2010. This database is a comprehensive data storage system that stores patient information including demographics, patient characteristics, perioperative management, and details regarding complications [7]. A query-building tool called Data Discovery and Query Builder (DDQB) was used to search the Mayo database and interrogate data files (International Business Machines, Corp.). The primary outcome variable was the presence of PTPS documented within 2 months of the surgery date. Among the cohort of thoracic surgical patients, cases of PTPS were identified using the following two approaches: (1) search for International Classification of Diseases-9 code for PTPS (code 338.22) and (2) free-text query of all notes in the electronic medical record for the following terms: thoracotomy pain, thoracotomy pain syndrome, postthoracotomy syndrome, postthoracotomy pain, postthoracotomy pain syndrome, post-thoracotomy syndrome, post-thoracotomy pain, post-thoracotomy pain syndrome, post thoracotomy syndrome, post thoracotomy pain, and post thoracotomy pain syndrome. The medical records of patients identified as having one or more key words were then manually reviewed to confirm eligibility. Sixty-six patients had a thoracotomy procedure with an epidural catheter used for perioperative analgesia and were seen in the Pain Clinic 2 months or greater after their thoracotomy for ipsilateral PTPS. In cases of repeat thoracotomies, only the date of the patient's most recent thoracotomy was used to calculate the minimum 2-month interval between thoracotomy and first documentation of PTPS. Identification of false positive cases was minimized by excluding those cases identified as “no thoracotomy pain” and “denies thoracotomy pain.” For each case of PTPS, a control patient who underwent thoracotomy and had their analgesia managed by a dedicated acute pain service utilizing an epidural catheter was identified. The control group patients were never diagnosed with PTPS and were matched with PTPS cases by patient age, sex, surgeon, and date of surgery. The control group patients were limited to a local residence status using a tristate area (MN, IA, and WI) to increase their chances of presenting to the Mayo Clinic Rochester Pain Clinic should PTPS have developed. All control group patients had follow-up at Mayo beyond 2 months after thoracotomy and the diagnosis was never made.

The following information was collected from the medical record: age, gender, height, weight, PTPS, side of PTPS, date of Pain Clinic visit, date of surgery, side of surgery, surgeon, surgical procedure, medications taken at Pain Clinic visit, epidural catheter placement date and time, the thoracic spinal level at which the epidural catheter was placed, the loading dose of opioid, whether the epidural had to be replaced, when the epidural was removed, why the epidural was removed, the epidural infusion rate and medications, and the dose of pain medications administered. Numeric rating scores (NRS) were collected every 4 hours of pain at rest and with movement (including coughing).

Clinical records were reviewed by the investigators on the study. Data were collected from the Mayo electronic medical record using a standardized data collection form and were then entered into the REDCap (Research Electronic Data Capture) electronic data capture tools at Mayo Clinic [9].

### 2.1. Statistical Analysis

Data were summarized and compared for the PTPS versus control groups. Categorical variables were compared using the Pearson chi-square test or Fisher's exact test, and continuous variables were compared using the two-sample *t*-test (or Rank Sum test). All analyses were two-tailed with a significance level of *P* < 0.05. All statistical analyses were conducted using JMP statistical software (version 9.0, SAS Institute Inc., Cary, North Carolina).

## 3. Results

Sixty-six patients were included in the postthoracotomy pain group, and sixty-six controls were matched for age, gender, surgeon, and date of surgery. There was no statistically significant difference between groups in terms of age, body mass index, gender, procedure type, or thoracotomy side (Table 1).

Overall, the age of the cohorts was 59 years, and 68 patients were male and 64 patients were female. Of the 64 females, 32 developed PTPS and 32 did not. Of the 68 males, 34 developed PTPS and 34 did not. There was no statistically significant difference between procedure types and the subsequent development of PTPS (*P* < 0.54, Pearson). Further, there was no statistical difference between patients who underwent more than one thoracic surgery and the subsequent development of PTPS (9 control patients (14%) and 16 PTPS patients (24%) underwent more than one thoracic surgery, *P* < 0.12, Pearson).

The median hospital duration was significantly different between control patients and PTPS patients. Control patients' median (IQR) hospital stay was 5 days (4, 6) compared with PTPS patients' stay of 6 days (5, 8), *P* < 0.020 (*t*-test). The median duration for patients having indwelling chest tubes was not significantly different between control patients and PTPS patients. Control patients' median (IQR) chest tube duration was 5 days (4, 6) compared with PTPS patients' chest tube duration of 5 days (4, 7), *P* < 0.069 (*t*-test).

The total opioid equivalent utilized for the first three days postoperatively was significantly different between control patients and PTPS patients. The median (IQR) total oral morphine equivalent utilized was 236.5 mg (73, 508) for controls and 365.5 mg (115.6, 874) for PTPS patients (*P* < 0.005, *t*-test) (Table 2).

Postoperative pain scores are summarized in Table 3. Only on POD1 did PTPS patients experience more pain with movement than control patients (*P* < 0.03). Otherwise, average reported pain scores were not significantly different between groups.

There was no statistically significant difference between groups in characteristics of epidural analgesia management. The median epidural level in the PTPS group and the control group was T6-7 (*P* < 0.70, Pearson). For the control group, the interquartile range was T5-6 and T8-9. For the PTPS group, the interquartile range was T5-6 and T7-8. Fifty-six patients (85%) in the control group had thoracic epidural catheters, and ten patients (15%) had lumbar epidural catheters. Fifty-seven patients (86%) in the PTPS group had thoracic epidural catheters, and nine patients (14%) had lumbar epidural catheters. There was also no statistically significant difference between groups in the type of opioid used for initial epidural loading dose (*P* < 0.58, Wilcoxon). Overall, 69 (52%) of patients received hydromorphone, 26 (20%) received fentanyl, and 13 (10%) received morphine for their epidural opioid loading dose, while 22 patients (17%) did not receive an opioid for an epidural loading dose. There was no difference in the selection of opioid medication or infusion used for epidural management, and there was no statistically significant difference between the initial epidural infusion type and the subsequent development of PTPS (*P* < 0.19, Pearson). The two most common initial epidural infusions were hydromorphone 10 mcg/mL with bupivacaine 0.075% (50 patients, 38%) and fentanyl 5 mcg/mL with bupivacaine 0.075% (30 patients, 23%). There was no statistically significant difference between groups in epidural catheter duration (*P* < 0.06, Wilcoxon Rank Sum). The median epidural catheter duration in the control group was 2.8 days (2.4–3.0, interquartile range) and was 2.9 days (2.7–3.0, interquartile range) in the PTPS group.

Median duration of last documentation of the 66 PTPS patients' diagnosis was 17 months (10–31 months, 25–75% IQR). At the time of follow-up, 13 patients (20%) were not taking any analgesic medications. Twenty-eight patients (42%) were taking one analgesic medication, 18 patients (27%) were taking two medications, and seven patients (11%) were taking three medications. Opioids were being utilized by 32 patients (48%). Antineuropathic pain medications (such as gabapentin, lidocaine transdermal patches, duloxetine, carbamazepine, and tricyclic antidepressants) were also being utilized by 32 patients (48%). Acetaminophen was utilized by eight patients (12%), and nonsteroidal anti-inflammatory medications were utilized by 13 patients (20%).

## 4. Discussion

Chronic PTPS remains a significant problem, despite aggressive postoperative analgesia [1, 2, 4, 10–13]. Postthoracotomy pain syndrome decreases quality of life with decreased physical function, decreased vitality, and ongoing consumption of analgesics [1, 2]. Chronic PTPS has proven to be very challenging to prevent, as it develops even following continuous thoracic epidural analgesia managed by a dedicated acute pain service utilizing multimodal adjuvant therapies including acetaminophen, nonsteroidal analgesics, opioids, and gabapentin [1, 2]. Of note, perioperative gabapentin has not been shown to prevent PTPS in prospective, randomized, controlled, and registered studies [2, 14], and gabapentinoids result in postoperative sedation and respiratory depression [15–17]. Genetic differences have not been identified as a risk factor in the development of chronic postsurgical pain [18]. Prolonged hospitalization and greater acute opioid usage are predictors for PTPS and therefore can be utilized as potential signals that a patient might benefit from closer follow-up and early referral to pain management specialists. Continued advances in surgical techniques, less traumatic chest drainage and suction equipment, and intercostal injections of liposomal local anesthetic preparations in high-risk patients may be valuable future studies to begin decreasing the incidence and severity of PTPS.

This case-control study of patients detected that the median hospital duration was significantly longer for PTPS patients. This raises the question of which contributing surgical or medical issues not only prolong hospitalization but are potentially associated with increased risk for the development of PTPS. Some preexisting medical conditions such as chronic obstructive pulmonary disease and/or postoperative complications such as infections, air or chyle leaks, and reoperations can lead to prolonged hospitalizations and prolonged causes of pain. Chest tubes are utilized following thoracotomy to evacuate air and fluid from the pleural space and promote lung expansion [19]. The presence of a chest tube is associated with an increased risk of developing PTPS [20]. The duration of chest tube drainage has also been shown to be a risk factor for the subsequent development of PTPS [21]. Chest tubes typically remain indwelling longer with prolonged chyle leaks, empyemas, air leaks, and hemothoraces and cause significant acute postoperative pain that can be challenging to treat [22]. Chest tube duration was not statistically significantly different between control and PTPS groups in this study, however.

While our thoracic epidural management and multimodal analgesic regimen produced statistically similar acute postoperative pain scores overall, the PTPS patients' opioid consumption was greater. The total opioid equivalent utilized for the first three days postoperatively was significantly greater in the PTPS patients. This suggests that patients who subsequently developed PTPS needed more opioid in the first three postoperative days and may have had greater acute pain that resulted in greater opioid usage.

We did not observe a significant difference in average rest pain or average movement pain between PTPS and control groups. A dedicated inpatient pain service managed the epidural infusions and other analgesics and helped achieve appropriate analgesia. Some authors have noted significant differences in acute postoperative pain in patients who have subsequently developed PTPS. Şentürk et al. [10] noted that 83% of patients having pain NRS ≥ 2 on the second postoperative day subsequently reported having PTPS at 6 months. This compared with 48% of patients who had NRS ≤ 2 on the second postoperative day who subsequently developed PTPS at 6 months. Ochroch et al. [4] also noted that acute pain at the first postoperative day was predictive of pain 24 weeks after surgery, but not 36 or 48 weeks after surgery. Katz et al. [23] observed virtually identical acute postoperative patient-controlled morphine usage between patients who subsequently had PTPS at 1.5 years and those who did not. Of note, epidural analgesia was not provided in the study by Katz et al. In our previous study [1], we did not note any significant difference between pain scores at rest or with coughing on the first or second morning after surgery between PTPS patients and control patients. Katz et al. found that the severity of acute postoperative pain correlated with the severity of chronic postthoracotomy pain. Pain intensity at rest and after movement on the first day after surgery was significantly greater in patients who subsequently had PTPS 1.5 years later [23]. Gotoda et al. found that early acute postoperative pain increased the probability of developing postthoracotomy pain syndrome [24], and Wang et al. [21] found that more intense wound pain predicted PTPS.

Grider et al. [25] observed that thoracic epidural analgesia (TEA) with bupivacaine and hydromorphone may improve acute postoperative analgesia compared with TEA with bupivacaine alone or paravertebral infusion alone. Epidural administration of bupivacaine and hydromorphone has been previously described [2, 26–29] and can provide significant postoperative analgesia but unfortunately has not been shown to decrease the incidence of PTPS [25].

The procedure type, thoracotomy side, repeat thoracic surgery, and the surgeon did not predict PTPS. In our previous study [1], we also did not observe a significant difference between surgical sides and PTPS. In contrast, Richardson et al. [20] found that resection of a rib decreased the probability of developing PTPS, and the left 8th intercostal incision for antireflux procedures increased the risk of developing PTPS. Ivor Lewis surgery did not change the risk of developing PTPS in Richardson's study, though.

This study is retrospective and is therefore limited in terms of the strength of our conclusions. The possibility of a type I error also exists. Preoperative opioid use was not an exclusion criterion and might have affected the total opioid utilized by a patient perioperatively. Numerous providers staff the inpatient pain service and selection of the type and rate of epidural infusion was likely subject to individual preferences. Pain scores with movement were not solicited as consistently in the beginning of our 11-year retrospective study period, and this reflects our change in clinical practice to attend to both rest and movement pain scores as a more thorough assessment of patient comfort. Our data is subject to the completeness of the record, but we utilized experienced abstractors. We limited the cases to local patients who were residents in the tristate area to maximize the likelihood of follow-up by the surgeons and referral to the Mayo Clinic Rochester Pain Clinic, and all patients were followed up at Mayo beyond 2 months after thoracotomy.

## 5. Conclusions

We observed that the median hospital duration was significantly different between control patients and PTPS patients. This risk factor has not been previously reported and provides a simple marker for patients who might be at increased risk for the development of PTPS. Similarly, the total opioid equivalent utilized in oral morphine mg equivalents for the first three days postoperatively was significantly different between control patients and PTPS patients. Greater opioid usage postoperatively is also a simple marker for identifying patients who might be at risk for PTPS. Early referral to pain management specialists can then occur in these patients with increased risk for PTPS.

## Figures and Tables

**Table 1:** Patient and procedural characteristics.

Characteristic	PTPS (*N* = 66)	Control (*N* = 66)
*Age* (years, mean ± SD)	59.0 ± 11.7	59.2 ± 13.1
*BMI* (kg/m^2^, mean ± SD)	27.4 ± 5.1	28.7 ± 6.8
*Gender*		
Male (*n*, %)	34 (52%)	34 (52%)
Female (*n*, %)	32 (48%)	32 (48%)
*Procedure*		
Pneumonectomy	8 (12%)	7 (11%)
Other thoracotomies	52 (79%)	55 (83%)
Combined thoracotomy and laparotomy	6 (9%)	4 (6%)
*Thoracotomy side* ^*∗*^		
Left	33 (50%)	25 (38%)
Right	30 (45%)	41 (62%)
Bilateral	3 (5%)	0 (0%)

There were no statistically significant differences between groups. Statistical analyses were conducted using Student's *t*-test for continuous variables and Pearson chi-square test for categorical variables. ^*∗*^ *P* = 0.05.

**Table 2:** Postoperative analgesic outcomes.

Characteristic	PTPS (*N* = 66)	Control (*N* = 66)	*P* value
*Total opioid equivalent* ^*∗*^			0.005
Median (25, 75 IQ)	366 (116, 874)	237 (74, 508)	
*Type of epidural opioid infusion*			0.367
*Fentanyl*	17 (26%)	14 (21%)	
*Hydromorphone*	44 (67%)	42 (64%)	
*Morphine*	5 (8%)	10 (15%)	
*Epidural bupivacaine infusion*	59 (89%)	55 (83%)	0.448

*P* values are from Student's *t*-test.

^*∗*^Opioid equivalents were calculated as oral morphine equivalents (mg) during the first three postoperative days and did not include epidural opioid [8].

**Table 3:** Postoperative pain scores (NRS).

Characteristic	PTPS		Control		*P* value
	*N*	Mean ± SD	*N*	Mean ± SD	
*Rest pain*					
POD1	33	4.7 ± 2.8	33	4.0 ± 3.1	0.30
POD2	37	4.1 ± 2.6	37	3.4 ± 2.3	0.25
POD3	31	2.9 ± 2.2	31	2.4 ± 2.3	0.38
Overall	44	4.1 ± 2.2	47	3.6 ± 2.6	0.38

*Movement pain*					
POD1	9	6.0 ± 2.5	10	3.3 ± 2.2	0.03
POD2	20	6.4 ± 2.8	26	6.1 ± 2.9	0.74
POD3	16	6.1 ± 3.1	24	5.1 ± 2.7	0.32
Overall	25	6.2 ± 2.5	30	5.0 ± 2.4	0.08

^*∗*^ *P* values are from ANOVA.

## References

[1] Kinney M. A. O., Hooten W. M., Cassivi S. D. (2012). Chronic postthoracotomy pain and health-related quality of life. *Annals of Thoracic Surgery*.

[2] Kinney M. A. O., Mantilla C. B., Carns P. E. (2012). Preoperative gabapentin for acute post-thoracotomy analgesia: a randomized, double-blinded, active placebo-controlled study. *Pain Practice*.

[3] Ochroch E. A., Gottschalk A., Troxel A. B., Farrar J. T. (2006). Women suffer more short and long-term pain than men after major thoracotomy. *Clinical Journal of Pain*.

[4] Ochroch E. A., Gottschalk A., Augostides J. (2002). Long-term pain and activity during recovery from major thoracotomy using thoracic epidural analgesia. *Anesthesiology*.

[5] Wildgaard K., Ravn J., Kehlet H. (2009). Chronic post-thoracotomy pain: a critical review of pathogenic mechanisms and strategies for prevention. *European Journal of Cardio-Thoracic Surgery*.

[6] Joshi G. P., Bonnet F., Shah R. (2008). A systematic review of randomized trials evaluating regional techniques for postthoracotomy analgesia. *Anesthesia and Analgesia*.

[7] Chute C. G., Beck S. A., Fisk T. B., Mohr D. N. (2010). The Enterprise Data Trust at Mayo Clinic: a semantically integrated warehouse of biomedical data. *Journal of the American Medical Informatics Association*.

[8] Hanks G. W., Conno F. D., Cherny N. (2001). Morphine and alternative opioids in cancer pain: the EAPC recommendations. *British Journal of Cancer*.

[9] Harris P. A., Taylor R., Thielke R., Payne J., Gonzalez N., Conde J. G. (2009). Research electronic data capture (REDCap)-a metadata-driven methodology and workflow process for providing translational research informatics support. *Journal of Biomedical Informatics*.

[10] Şentürk M., Özcan P. E., Talu G. K. (2002). The effects of three different analgesia techniques on long-term postthoracotomy pain. *Anesthesia & Analgesia*.

[11] Bong C. L., Samuel M., Ng J. M., Ip-Yam C. (2005). Effects of preemptive epidural analgesia on post-thoracotomy pain. *Journal of Cardiothoracic and Vascular Anesthesia*.

[12] Tiippana E., Nilsson E., Kalso E. (2003). Post-thoracotomy pain after thoracic epidural analgesia: a prospective follow-up study. *Acta Anaesthesiologica Scandinavica*.

[13] Guastella V., Mick G., Soriano C. (2011). A prospective study of neuropathic pain induced by thoracotomy: incidence, clinical description, and diagnosis. *Pain*.

[14] Grosen K., Drewes A. M., Højsgaard A., Pfeiffer-Jensen M., Hjortdal V. E., Pilegaard H. K. (2014). Perioperative gabapentin for the prevention of persistent pain after thoracotomy: a randomized controlled trial. *European Journal of Cardio-thoracic Surgery*.

[15] Myhre M., Diep L. M., Stubhaug A. (2016). Pregabalin has analgesic, ventilatory, and cognitive effects in combination with remifentanil. *Anesthesiology*.

[16] Chelly J. E. (2013). Pregabalin effective for the prevention of chronic postsurgical pain: really?. *Anesthesia & Analgesia*.

[17] Weingarten T. N., Jacob A. K., Njathi C. W., Wilson G. A., Sprung J. (2015). Multimodal analgesic protocol and postanesthesia respiratory depression during phase I recovery after total joint arthroplasty. *Regional Anesthesia and Pain Medicine*.

[18] Montes A., Roca G., Sabate S. (2015). Genetic and clinical factors associated with chronic postsurgical pain after hernia repair, hysterectomy, and thoracotomy: a two-year multicenter cohort study. *Anesthesiology*.

[19] Brunelli A., Cassivi S. D., Fibla J., Di Nunzio L. (2010). Pleural pressure immediately after pulmonary lobectomy: single versus double chest tubes for suction. *The Journal of Thoracic and Cardiovascular Surgery*.

[20] Richardson J., Sabanathan S., Mearns A. J., Sides C., Goulden C. P. (1994). Post-thoracotomy neuralgia. *The Pain Clinic*.

[21] Wang H.-T., Liu W., Luo A.-L., Ma C., Huang Y.-G. (2012). Prevalence and risk factors of chronic post-thoracotomy pain in Chinese patients from peking union medical college hospital. *Chinese Medical Journal*.

[22] Stanik-Hutt J. A., Soeken K. L., Belcher A. E., Fontaine D. K., Gift A. G. (2001). Pain experiences of traumatically injured patients in a critical care setting. *American Journal of Critical Care*.

[23] Katz J., Jackson M., Kavanagh B. P., Sandler A. N. (1996). Acute pain after thoracic surgery predicts long-term post-thoracotomy pain. *Clinical Journal of Pain*.

[24] Gotoda Y., Kambara N., Sakai T., Kishi Y., Kodama K., Koyama T. (2001). The morbidity, time course and predictive factors for persistent post-thoracotomy pain. *European Journal of Pain*.

[25] Grider J. S., Mullet T. W., Saha S. P., Harned M. E., Sloan P. A. (2012). A randomized, double-blind trial comparing continuous thoracic epidural bupivacaine with and without opioid in contrast to a continuous paravertebral infusion of bupivacaine for post-thoracotomy pain. *Journal of Cardiothoracic & Vascular Anesthesia*.

[26] Sinatra R. S., Levin S., Ocampo C. A. (2000). Neuroaxial hydromorphone for control of postsurgical, obstetric, and chronic pain. *Seminars in Anesthesia*.

[27] Sinatra R. S., Eige S., Chung J. H. (2002). Continuous epidural infusion of 0.05% bupivacaine plus hydromorphone for labor analgesia: an observational assessment in 1830 parturients. *Anesthesia & Analgesia*.

[28] Liu S. S., Bieltz M., Wukovits B., John R. S. (2010). Prospective survey of patient-controlled epidural analgesia with bupivacaine and hydromorphone in 3736 postoperative orthopedic patients. *Regional Anesthesia & Pain Medicine*.

[29] Brown M. J., Kor D. J., Allen M. S. (2013). Dual-epidural catheter technique and perioperative outcomes after ivor-lewis esophagectomy. *Regional Anesthesia and Pain Medicine*.

